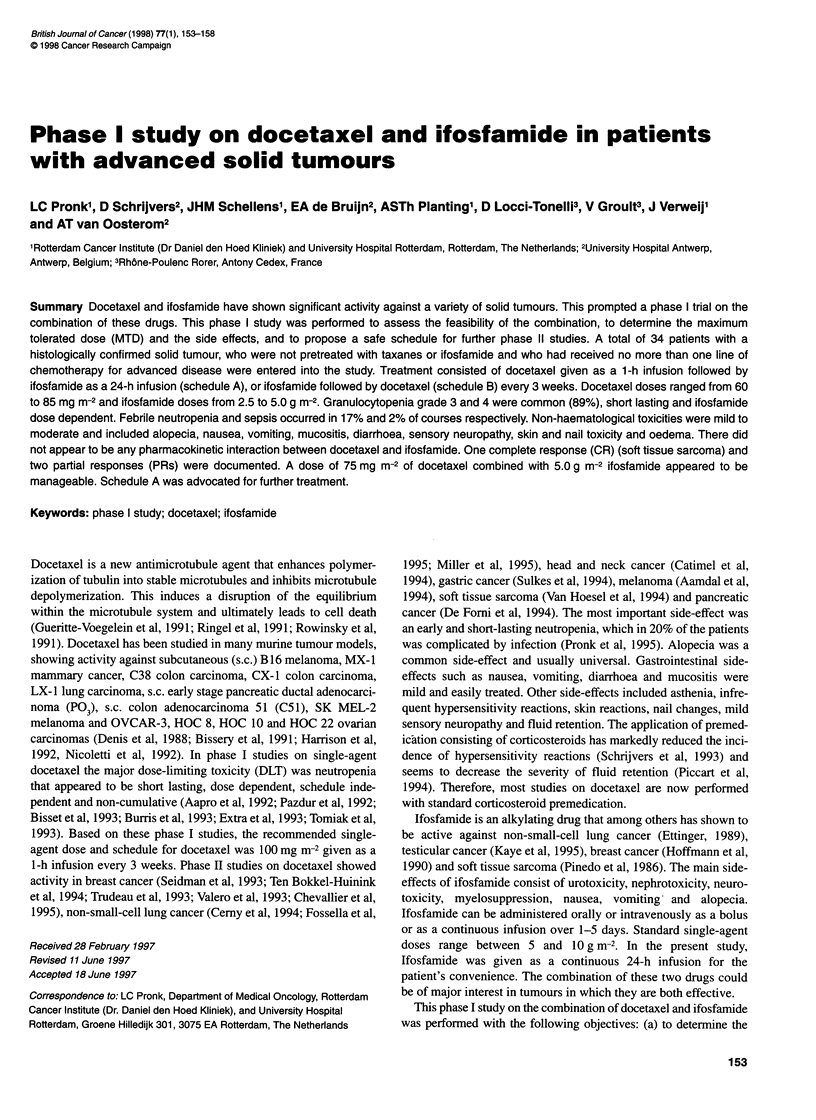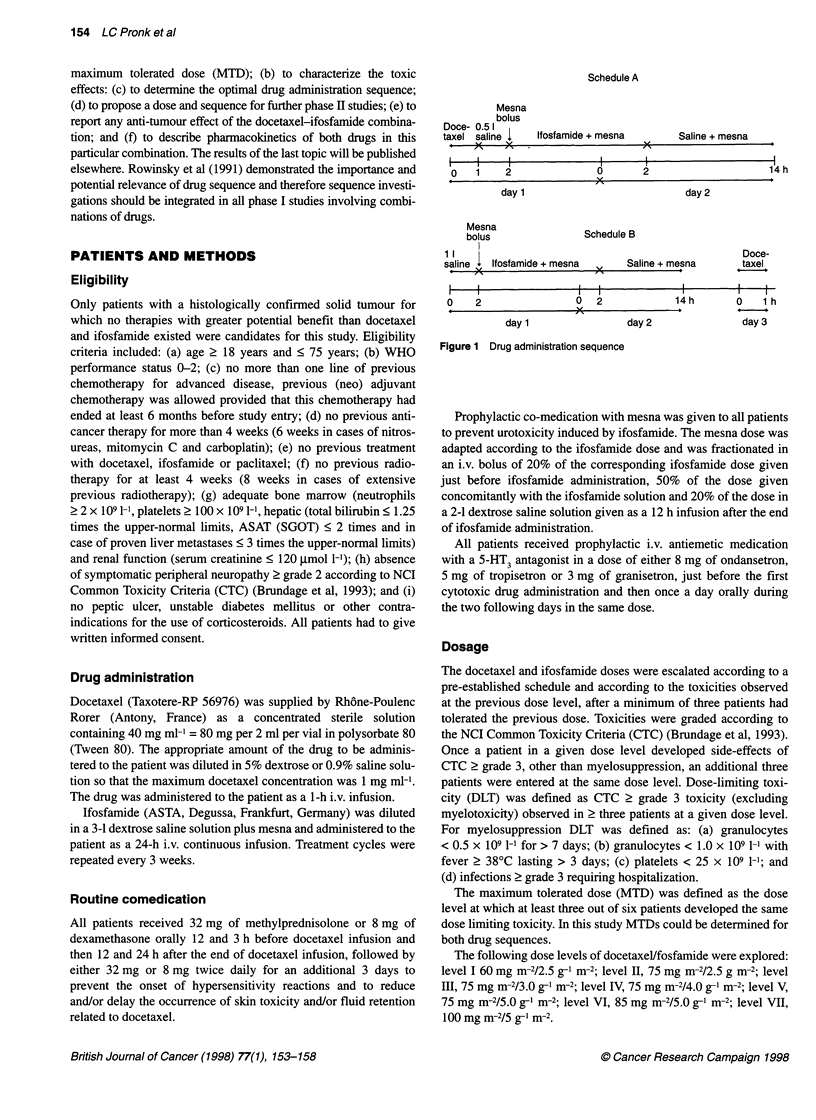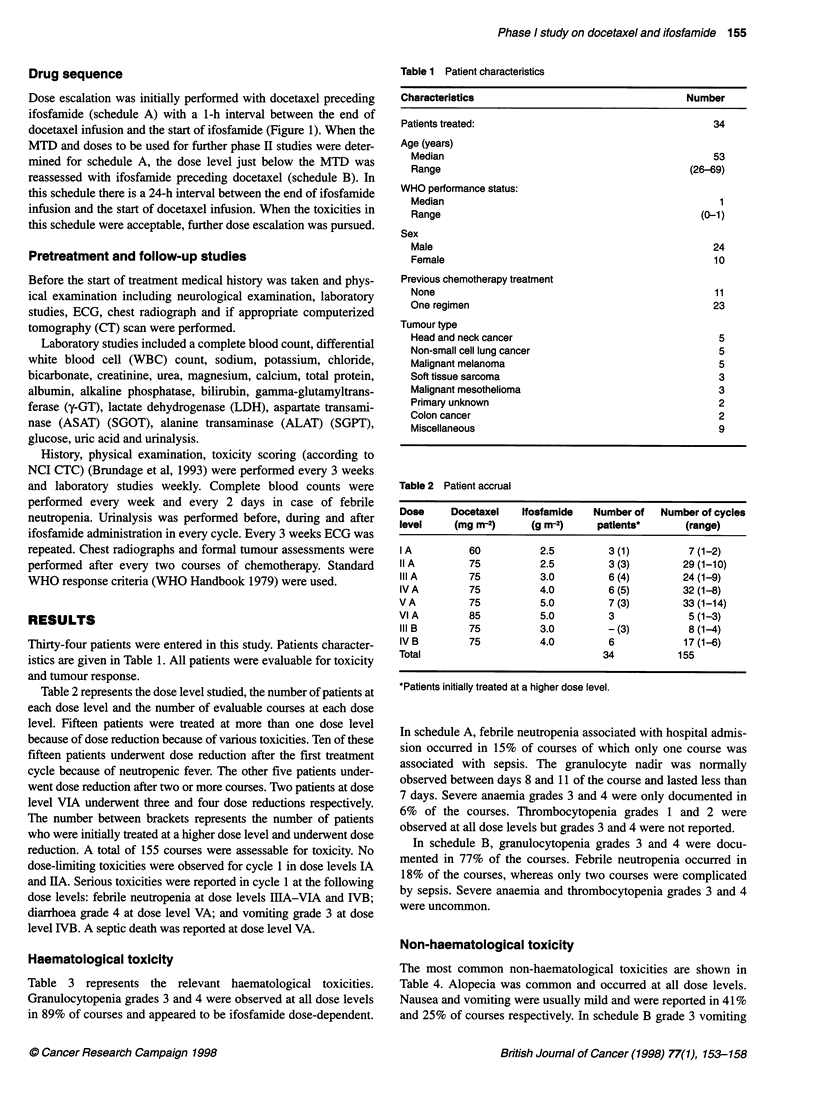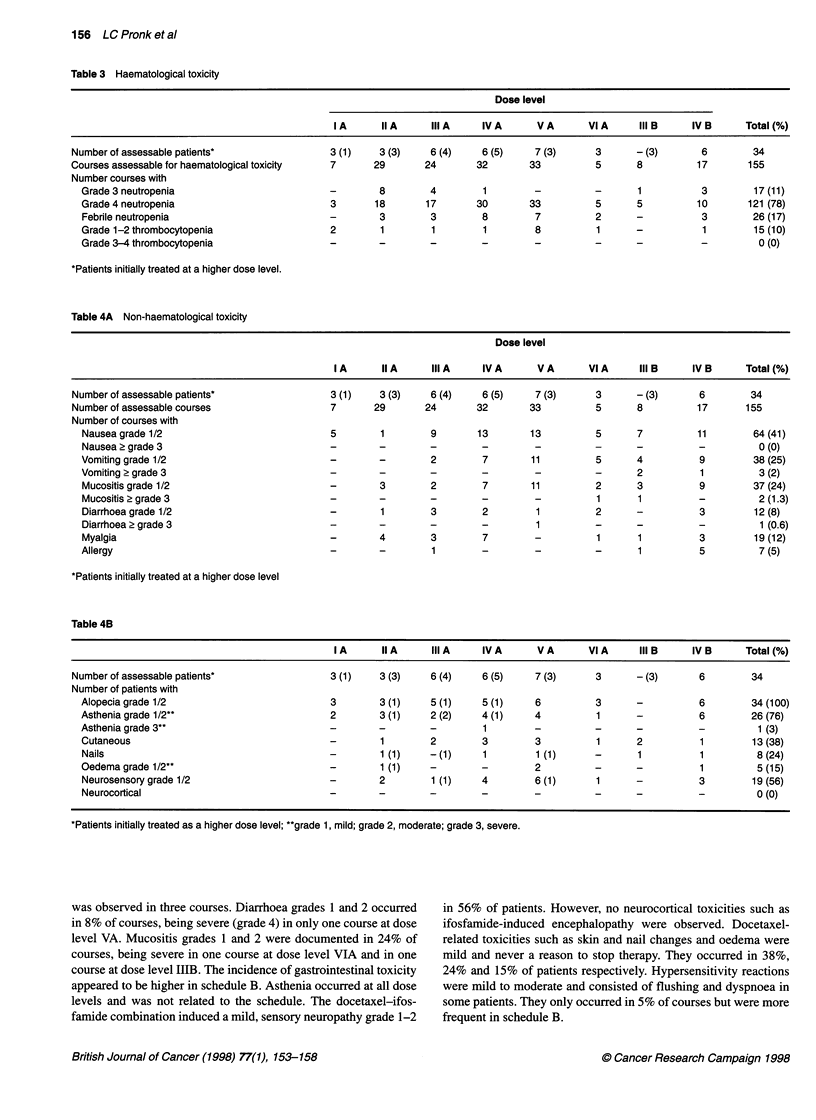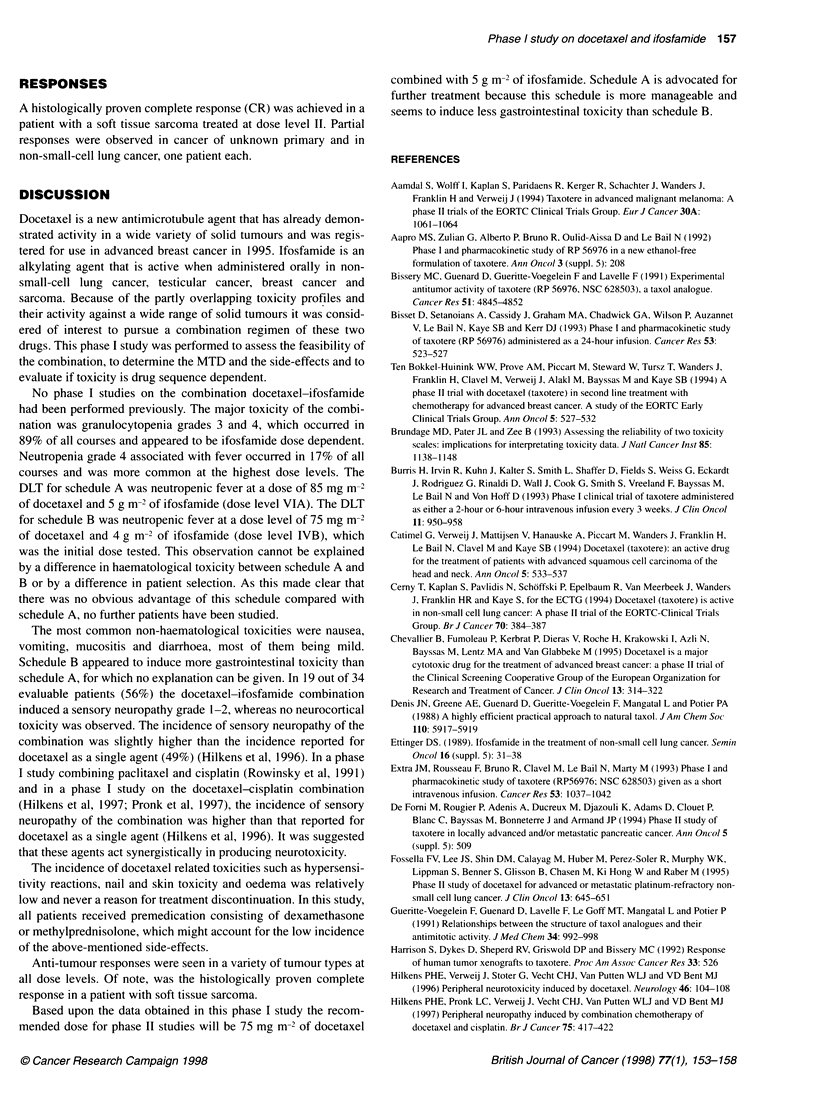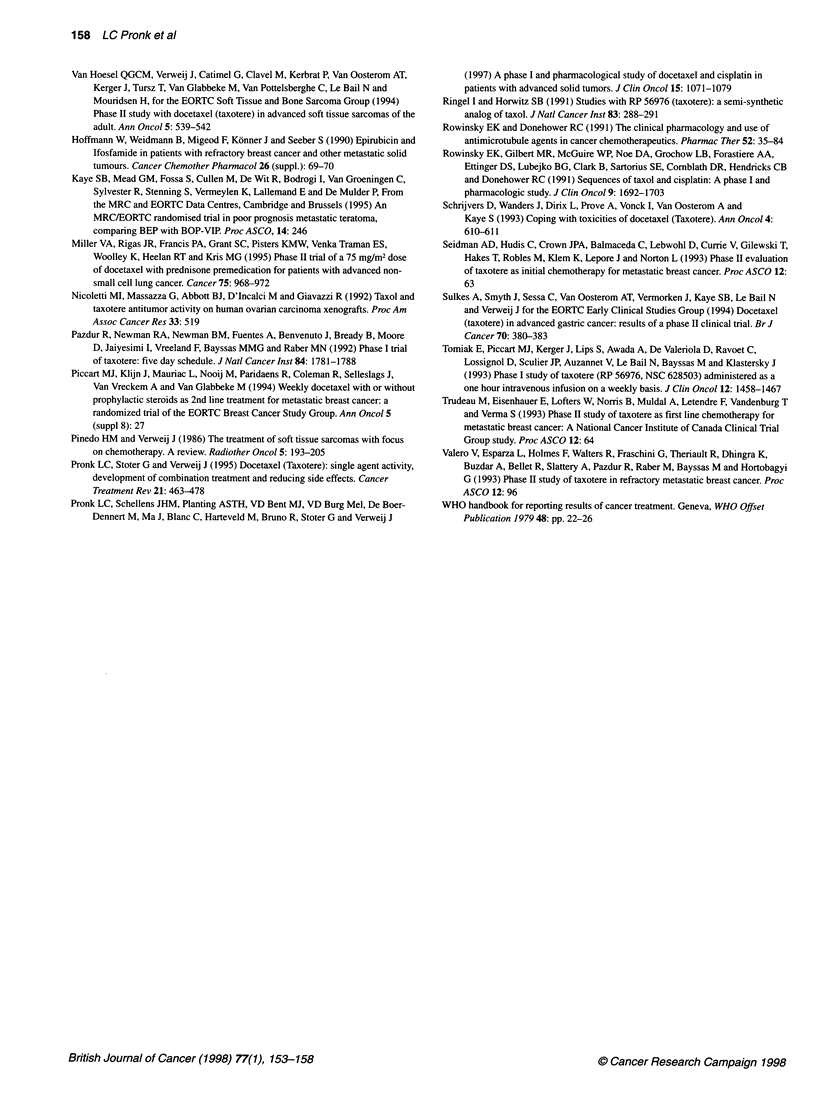# Phase I study on docetaxel and ifosfamide in patients with advanced solid tumours.

**DOI:** 10.1038/bjc.1998.24

**Published:** 1998

**Authors:** L. C. Pronk, D. Schrijvers, J. H. Schellens, E. A. de Bruijn, A. S. Planting, D. Locci-Tonelli, V. Groult, J. Verweij, A. T. van Oosterom

**Affiliations:** Rotterdam Cancer Institute (Dr Daniel den Hoed Kliniek) and University Hospital Rotterdam, The Netherlands.

## Abstract

Docetaxel and ifosfamide have shown significant activity against a variety of solid tumours. This prompted a phase I trial on the combination of these drugs. This phase I study was performed to assess the feasibility of the combination, to determine the maximum tolerated dose (MTD) and the side effects, and to propose a safe schedule for further phase II studies. A total of 34 patients with a histologically confirmed solid tumour, who were not pretreated with taxanes or ifosfamide and who had received no more than one line of chemotherapy for advanced disease were entered into the study. Treatment consisted of docetaxel given as a 1-h infusion followed by ifosfamide as a 24-h infusion (schedule A), or ifosfamide followed by docetaxel (schedule B) every 3 weeks. Docetaxel doses ranged from 60 to 85 mg m(-2) and ifosfamide doses from 2.5 to 5.0 g m(-2). Granulocytopenia grade 3 and 4 were common (89%), short lasting and ifosfamide dose dependent. Febrile neutropenia and sepsis occurred in 17% and 2% of courses respectively. Non-haematological toxicities were mild to moderate and included alopecia, nausea, vomiting, mucositis, diarrhoea, sensory neuropathy, skin and nail toxicity and oedema. There did not appear to be any pharmacokinetic interaction between docetaxel and ifosfamide. One complete response (CR) (soft tissue sarcoma) and two partial responses (PRs) were documented. A dose of 75 mg m(-2) of docetaxel combined with 5.0 g m(-2) ifosfamide appeared to be manageable. Schedule A was advocated for further treatment.


British Journal of Cancer (1998) 77(1), 153–158
© 1998 Cancer Research Campaign

### Phase I study on docetaxel and ifosfamide in patients with advanced solid tumours

**LC Pronk[1], D Schrijvers[2], JHM Schellens[1], EA de Bruijn[2], ASTh Planting[1], D Locci-Tonelli[3], V Groult[3], J Verweij[1] and AT van Oosterom[2]**

[1]Rotterdam Cancer Institute (Dr Daniel den Hoed Kliniek) and University Hospital Rotterdam, Rotterdam, The Netherlands; [2]University Hospital Antwerp, Antwerp, Belgium; [3]Rhône-Poulenc Rorer, Antony Cedex, France

**Summary** Docetaxel and ifosfamide have shown significant activity against a variety of solid tumours. This prompted a phase I trial on the combination of these drugs. This phase I study was performed to assess the feasibility of the combination, to determine the maximum tolerated dose (MTD) and the side effects, and to propose a safe schedule for further phase II studies. A total of 34 patients with a histologically confirmed solid tumour, who were not pretreated with taxanes or ifosfamide and who had received no more than one line of chemotherapy for advanced disease were entered into the study. Treatment consisted of docetaxel given as a 1-h infusion followed by ifosfamide as a 24-h infusion (schedule A), or ifosfamide followed by docetaxel (schedule B) every 3 weeks. Docetaxel doses ranged from 60 to 85 mg $m^{-2}$ and ifosfamide doses from 2.5 to 5.0 g $m^{-2}$. Granulocytopenia grade 3 and 4 were common (89%), short lasting and ifosfamide dose dependent. Febrile neutropenia and sepsis occurred in 17% and 2% of courses respectively. Non-haematological toxicities were mild to moderate and included alopecia, nausea, vomiting, mucositis, diarrhoea, sensory neuropathy, skin and nail toxicity and oedema. There did not appear to be any pharmacokinetic interaction between docetaxel and ifosfamide. One complete response (CR) (soft tissue sarcoma) and two partial responses (PRs) were documented. A dose of 75 mg $m^{-2}$ of docetaxel combined with 5.0 g $m^{-2}$ ifosfamide appeared to be manageable. Schedule A was advocated for further treatment.

**Keywords:** phase I study; docetaxel; ifosfamide

Docetaxel is a new antimicrotubule agent that enhances polymerization of tubulin into stable microtubules and inhibits microtubule depolymerization. This induces a disruption of the equilibrium within the microtubule system and ultimately leads to cell death (Guerite-Voegelein et al, 1991; Ringel et al, 1991; Rowinsky et al, 1991). Docetaxel has been studied in many murine tumour models, showing activity against subcutaneous (s.c.) B16 melanoma, MX-1 mammary cancer, C38 colon carcinoma, CX-1 colon carcinoma, LX-1 lung carcinoma, s.c. early stage pancreatic ductal adenocarcinoma ($PO_3$), s.c. colon adenocarcinoma 51 (C51), SK MEL-2 melanoma and OVCAR-3, HOC 8, HOC 10 and HOC 22 ovarian carcinomas (Denis et al, 1988; Bissery et al, 1991; Harrison et al, 1992, Nicoletti et al, 1992). In phase I studies on single-agent docetaxel the major dose-limiting toxicity (DLT) was neutropenia that appeared to be short lasting, dose dependent, schedule independent and non-cumulative (Aapro et al, 1992; Pazdur et al, 1992; Bisset et al, 1993; Burris et al, 1993; Extra et al, 1993; Tomiak et al, 1993). Based on these phase I studies, the recommended single-agent dose and schedule for docetaxel was 100 mg $m^{-2}$ given as a 1-h infusion every 3 weeks. Phase II studies on docetaxel showed activity in breast cancer (Seidman et al, 1993; Ten Bokkel-Huinink et al, 1994; Trudeau et al, 1993; Valero et al, 1993; Chevallier et al, 1995), non-small-cell lung cancer (Cerny et al, 1994; Fossella et al, 1995; Miller et al, 1995), head and neck cancer (Catimel et al, 1994), gastric cancer (Sulkes et al, 1994), melanoma (Aamdal et al, 1994), soft tissue sarcoma (Van Hoesel et al, 1994) and pancreatic cancer (De Forni et al, 1994). The most important side-effect was an early and short-lasting neutropenia, which in 20% of the patients was complicated by infection (Pronk et al, 1995). Alopecia was a common side-effect and usually universal. Gastrointestinal side-effects such as nausea, vomiting, diarrhoea and mucositis were mild and easily treated. Other side-effects included asthenia, infrequent hypersensitivity reactions, skin reactions, nail changes, mild sensory neuropathy and fluid retention. The application of premedication consisting of corticosteroids has markedly reduced the incidence of hypersensitivity reactions (Schrijvers et al, 1993) and seems to decrease the severity of fluid retention (Piccart et al, 1994). Therefore, most studies on docetaxel are now performed with standard corticosteroid premedication.

Received 28 February 1997
Revised 11 June 1997
Accepted 18 June 1997

Correspondence to: LC Pronk, Department of Medical Oncology, Rotterdam Cancer Institute (Dr. Daniel den Hoed Kliniek), and University Hospital Rotterdam, Groene Hilledijk 301, 3075 EA Rotterdam, The Netherlands

Ifosfamide is an alkylating drug that among others has shown to be active against non-small-cell lung cancer (Ettinger, 1989), testicular cancer (Kaye et al, 1995), breast cancer (Hoffmann et al, 1990) and soft tissue sarcoma (Pinedo et al, 1986). The main side-effects of ifosfamide consist of urotoxicity, nephrotoxicity, neurotoxicity, myelosuppression, nausea, vomiting and alopecia. Ifosfamide can be administered orally or intravenously as a bolus or as a continuous infusion over 1–5 days. Standard single-agent doses range between 5 and 10 g $m^{-2}$. In the present study, Ifosfamide was given as a continuous 24-h infusion for the patient's convenience. The combination of these two drugs could be of major interest in tumours in which they are both effective.

This phase I study on the combination of docetaxel and ifosfamide was performed with the following objectives: (a) to determine the maximum tolerated dose (MTD); (b) to characterize the toxic effects: (c) to determine the optimal drug administration sequence; (d) to propose a dose and sequence for further phase II studies; (e) to report any anti-tumour effect of the docetaxel–ifosfamide combination; and (f) to describe pharmacokinetics of both drugs in this particular combination. The results of the last topic will be published elsewhere. Rowinsky et al (1991) demonstrated the importance and potential relevance of drug sequence and therefore sequence investigations should be integrated in all phase I studies involving combinations of drugs.

### PATIENTS AND METHODS

### Eligibility

Only patients with a histologically confirmed solid tumour for which no therapies with greater potential benefit than docetaxel and ifosfamide existed were candidates for this study. Eligibility criteria included: (a) age $\geq$ 18 years and $\leq$ 75 years; (b) WHO performance status 0–2; (c) no more than one line of previous chemotherapy for advanced disease, previous (neo) adjuvant chemotherapy was allowed provided that this chemotherapy had ended at least 6 months before study entry; (d) no previous anticancer therapy for more than 4 weeks (6 weeks in cases of nitrosureas, mitomycin C and carboplatin); (e) no previous treatment with docetaxel, ifosfamide or paclitaxel; (f) no previous radiotherapy for at least 4 weeks (8 weeks in cases of extensive previous radiotherapy); (g) adequate bone marrow (neutrophils $\geq 2 \times 10^9$ l$^{-1}$, platelets $\geq 100 \times 10^9$ l$^{-1}$, hepatic (total bilirubin $\leq$ 1.25 times the upper-normal limits, ASAT (SGOT) $\leq$ 2 times and in case of proven liver metastases $\leq$ 3 times the upper-normal limits) and renal function (serum creatinine $\leq$ 120 μmol l$^{-1}$); (h) absence of symptomatic peripheral neuropathy $\geq$ grade 2 according to NCI Common Toxicity Criteria (CTC) (Brundage et al, 1993); and (i) no peptic ulcer, unstable diabetes mellitus or other contra-indications for the use of corticosteroids. All patients had to give written informed consent.

### Drug administration

Docetaxel (Taxotere-RP 56976) was supplied by Rhône-Poulenc Rorer (Antony, France) as a concentrated sterile solution containing 40 mg ml$^{-1}$ = 80 mg per 2 ml per vial in polysorbate 80 (Tween 80). The appropriate amount of the drug to be administered to the patient was diluted in 5% dextrose or 0.9% saline solution so that the maximum docetaxel concentration was 1 mg ml$^{-1}$. The drug was administered to the patient as a 1-h i.v. infusion.

Ifosfamide (ASTA, Degussa, Frankfurt, Germany) was diluted in a 3-l dextrose saline solution plus mesna and administered to the patient as a 24-h i.v. continuous infusion. Treatment cycles were repeated every 3 weeks.

### Routine comedication

All patients received 32 mg of methylprednisolone or 8 mg of dexamethasone orally 12 and 3 h before docetaxel infusion and then 12 and 24 h after the end of docetaxel infusion, followed by either 32 mg or 8 mg twice daily for an additional 3 days to prevent the onset of hypersensitivity reactions and to reduce and/or delay the occurrence of skin toxicity and/or fluid retention related to docetaxel.

**Figure 1** Drug administration sequence

Prophylactic co-medication with mesna was given to all patients to prevent urotoxicity induced by ifosfamide. The mesna dose was adapted according to the ifosfamide dose and was fractionated in an i.v. bolus of 20% of the corresponding ifosfamide dose given just before ifosfamide administration, 50% of the dose given concomitantly with the ifosfamide solution and 20% of the dose in a 2-l dextrose saline solution given as a 12 h infusion after the end of ifosfamide administration.

All patients received prophylactic i.v. antiemetic medication with a 5-$HT_3$ antagonist in a dose of either 8 mg of ondansetron, 5 mg of tropisetron or 3 mg of granisetron, just before the first cytotoxic drug administration and then once a day orally during the two following days in the same dose.

### Dosage

The docetaxel and ifosfamide doses were escalated according to a pre-established schedule and according to the toxicities observed at the previous dose level, after a minimum of three patients had tolerated the previous dose. Toxicities were graded according to the NCI Common Toxicity Criteria (CTC) (Brundage et al, 1993). Once a patient in a given dose level developed side-effects of CTC $\geq$ grade 3, other than myelosuppression, an additional three patients were entered at the same dose level. Dose-limiting toxicity (DLT) was defined as CTC $\geq$ grade 3 toxicity (excluding myelotoxicity) observed in $\geq$ three patients at a given dose level. For myelosuppression DLT was defined as: (a) granulocytes $< 0.5 \times 10^9$ l$^{-1}$ for > 7 days; (b) granulocytes $< 1.0 \times 10^9$ l$^{-1}$ with fever $\geq$ 38°C lasting > 3 days; (c) platelets $< 25 \times 10^9$ l$^{-1}$; and (d) infections $\geq$ grade 3 requiring hospitalization.

The maximum tolerated dose (MTD) was defined as the dose level at which at least three out of six patients developed the same dose limiting toxicity. In this study MTDs could be determined for both drug sequences.

The following dose levels of docetaxel/fosfamide were explored: level I 60 mg m$^{-2}$/2.5 g$^{-1}$ m$^{-2}$; level II, 75 mg m$^{-2}$/2.5 g m$^{-2}$; level III, 75 mg m$^{-2}$/3.0 g$^{-1}$ m$^{-2}$; level IV, 75 mg m$^{-2}$/4.0 g$^{-1}$ m$^{-2}$; level V, 75 mg m$^{-2}$/5.0 g$^{-1}$ m$^{-2}$; level VI, 85 mg m$^{-2}$/5.0 g$^{-1}$ m$^{-2}$; level VII, 100 mg m$^{-2}$/5 g$^{-1}$ m$^{-2}$.

© Cancer Research Campaign 1998

### Drug sequence

Dose escalation was initially performed with docetaxel preceding ifosfamide (schedule A) with a 1-h interval between the end of docetaxel infusion and the start of ifosfamide (Figure 1). When the MTD and doses to be used for further phase II studies were determined for schedule A, the dose level just below the MTD was reassessed with ifosfamide preceding docetaxel (schedule B). In this schedule there is a 24-h interval between the end of ifosfamide infusion and the start of docetaxel infusion. When the toxicities in this schedule were acceptable, further dose escalation was pursued.

### Pretreatment and follow-up studies

Before the start of treatment medical history was taken and physical examination including neurological examination, laboratory studies, ECG, chest radiograph and if appropriate computerized tomography (CT) scan were performed.

Laboratory studies included a complete blood count, differential white blood cell (WBC) count, sodium, potassium, chloride, bicarbonate, creatinine, urea, magnesium, calcium, total protein, albumin, alkaline phosphatase, bilirubin, gamma-glutamyltransferase ($\gamma$-GT), lactate dehydrogenase (LDH), aspartate transaminase (ASAT) (SGOT), alanine transaminase (ALAT) (SGPT), glucose, uric acid and urinalysis.

History, physical examination, toxicity scoring (according to NCI CTC) (Brundage et al, 1993) were performed every 3 weeks and laboratory studies weekly. Complete blood counts were performed every week and every 2 days in case of febrile neutropenia. Urinalysis was performed before, during and after ifosfamide administration in every cycle. Every 3 weeks ECG was repeated. Chest radiographs and formal tumour assessments were performed after every two courses of chemotherapy. Standard WHO response criteria (WHO Handbook 1979) were used.

### RESULTS

Thirty-four patients were entered in this study. Patients characteristics are given in Table 1. All patients were evaluable for toxicity and tumour response.

Table 2 represents the dose level studied, the number of patients at each dose level and the number of evaluable courses at each dose level. Fifteen patients were treated at more than one dose level because of dose reduction because of various toxicities. Ten of these fifteen patients underwent dose reduction after the first treatment cycle because of neutropenic fever. The other five patients underwent dose reduction after two or more courses. Two patients at dose level VIA underwent three and four dose reductions respectively. The number between brackets represents the number of patients who were initially treated at a higher dose level and underwent dose reduction. A total of 155 courses were assessable for toxicity. No dose-limiting toxicities were observed for cycle 1 in dose levels IA and IIA. Serious toxicities were reported in cycle 1 at the following dose levels: febrile neutropenia at dose levels IIIA–VIA and IVB; diarrhoea grade 4 at dose level VA; and vomiting grade 3 at dose level IVB. A septic death was reported at dose level VA.

### Haematological toxicity

Table 3 represents the relevant haematological toxicities. Granulocytopenia grades 3 and 4 were observed at all dose levels in 89% of courses and appeared to be ifosfamide dose-dependent.

**Table 1** Patient characteristics

| Characteristics | Number |
|---|---|
| Patients treated: | 34 |
| Age (years) | |
| Median | 53 |
| Range | (26–69) |
| WHO performance status: | |
| Median | 1 |
| Range | (0–1) |
| Sex | |
| Male | 24 |
| Female | 10 |
| Previous chemotherapy treatment | |
| None | 11 |
| One regimen | 23 |
| Tumour type | |
| Head and neck cancer | 5 |
| Non-small cell lung cancer | 5 |
| Malignant melanoma | 5 |
| Soft tissue sarcoma | 3 |
| Malignant mesothelioma | 3 |
| Primary unknown | 2 |
| Colon cancer | 2 |
| Miscellaneous | 9 |

**Table 2** Patient accrual

| Dose level | Docetaxel (mg $m^{-2}$) | Ifosfamide (g $m^{-2}$) | Number of patients* | Number of cycles (range) |
|---|---|---|---|---|
| I A | 60 | 2.5 | 3 (1) | 7 (1–2) |
| II A | 75 | 2.5 | 3 (3) | 29 (1–10) |
| III A | 75 | 3.0 | 6 (4) | 24 (1–9) |
| IV A | 75 | 4.0 | 6 (5) | 32 (1–8) |
| V A | 75 | 5.0 | 7 (3) | 33 (1–14) |
| VI A | 85 | 5.0 | 3 | 5 (1–3) |
| III B | 75 | 3.0 | – (3) | 8 (1–4) |
| IV B | 75 | 4.0 | 6 | 17 (1–6) |
| Total | | | 34 | 155 |

*Patients initially treated at a higher dose level.

In schedule A, febrile neutropenia associated with hospital admission occurred in 15% of courses of which only one course was associated with sepsis. The granulocyte nadir was normally observed between days 8 and 11 of the course and lasted less than 7 days. Severe anaemia grades 3 and 4 were only documented in 6% of the courses. Thrombocytopenia grades 1 and 2 were observed at all dose levels but grades 3 and 4 were not reported.

In schedule B, granulocytopenia grades 3 and 4 were documented in 77% of the courses. Febrile neutropenia occurred in 18% of the courses, whereas only two courses were complicated by sepsis. Severe anaemia and thrombocytopenia grades 3 and 4 were uncommon.

### Non-haematological toxicity

The most common non-haematological toxicities are shown in Table 4. Alopecia was common and occurred at all dose levels. Nausea and vomiting were usually mild and were reported in 41% and 25% of courses respectively. In schedule B grade 3 vomiting

© Cancer Research Campaign 1998

**Table 3** Haematological toxicity

| | Dose level | | | | | | | | |
|---|---|---|---|---|---|---|---|---|---|
| | I A | II A | III A | IV A | V A | VI A | III B | IV B | Total (%) |
| Number of assessable patients* | 3 (1) | 3 (3) | 6 (4) | 6 (5) | 7 (3) | 3 | – (3) | 6 | 34 |
| Courses assessable for haematological toxicity | 7 | 29 | 24 | 32 | 33 | 5 | 8 | 17 | 155 |
| Number courses with | | | | | | | | | |
| Grade 3 neutropenia | – | 8 | 4 | 1 | – | – | 1 | 3 | 17 (11) |
| Grade 4 neutropenia | 3 | 18 | 17 | 30 | 33 | 5 | 5 | 10 | 121 (78) |
| Febrile neutropenia | – | 3 | 3 | 8 | 7 | 2 | – | 3 | 26 (17) |
| Grade 1–2 thrombocytopenia | 2 | 1 | 1 | 1 | 8 | 1 | – | 1 | 15 (10) |
| Grade 3–4 thrombocytopenia | – | – | – | – | – | – | – | – | 0 (0) |

*Patients initially treated at a higher dose level.

**Table 4A** Non-haematological toxicity

| | Dose level | | | | | | | | |
|---|---|---|---|---|---|---|---|---|---|
| | I A | II A | III A | IV A | V A | VI A | III B | IV B | Total (%) |
| Number of assessable patients* | 3 (1) | 3 (3) | 6 (4) | 6 (5) | 7 (3) | 3 | – (3) | 6 | 34 |
| Number of assessable courses | 7 | 29 | 24 | 32 | 33 | 5 | 8 | 17 | 155 |
| Number of courses with | | | | | | | | | |
| Nausea grade 1/2 | 5 | 1 | 9 | 13 | 13 | 5 | 7 | 11 | 64 (41) |
| Nausea ≥ grade 3 | – | – | – | – | – | – | – | – | 0 (0) |
| Vomiting grade 1/2 | – | – | 2 | 7 | 11 | 5 | 4 | 9 | 38 (25) |
| Vomiting ≥ grade 3 | – | – | – | – | – | – | 2 | 1 | 3 (2) |
| Mucositis grade 1/2 | – | 3 | 2 | 7 | 11 | 2 | 3 | 9 | 37 (24) |
| Mucositis ≥ grade 3 | – | – | – | – | – | 1 | 1 | – | 2 (1.3) |
| Diarrhoea grade 1/2 | – | 1 | 3 | 2 | 1 | 2 | – | 3 | 12 (8) |
| Diarrhoea ≥ grade 3 | – | – | – | – | 1 | – | – | – | 1 (0.6) |
| Myalgia | – | 4 | 3 | 7 | – | 1 | 1 | 3 | 19 (12) |
| Allergy | – | – | 1 | – | – | – | 1 | 5 | 7 (5) |

*Patients initially treated at a higher dose level

**Table 4B**

| | I A | II A | III A | IV A | V A | VI A | III B | IV B | Total (%) |
|---|---|---|---|---|---|---|---|---|---|
| Number of assessable patients* | 3 (1) | 3 (3) | 6 (4) | 6 (5) | 7 (3) | 3 | – (3) | 6 | 34 |
| Number of patients with | | | | | | | | | |
| Alopecia grade 1/2 | 3 | 3 (1) | 5 (1) | 5 (1) | 6 | 3 | – | 6 | 34 (100) |
| Asthenia grade 1/2** | 2 | 3 (1) | 2 (2) | 4 (1) | 4 | 1 | – | 6 | 26 (76) |
| Asthenia grade 3** | – | – | – | 1 | – | – | – | – | 1 (3) |
| Cutaneous | – | 1 | 2 | 3 | 3 | 1 | 2 | 1 | 13 (38) |
| Nails | – | 1 (1) | – (1) | 1 | 1 (1) | – | 1 | 1 | 8 (24) |
| Oedema grade 1/2** | – | 1 (1) | – | – | 2 | – | – | 1 | 5 (15) |
| Neurosensory grade 1/2 | – | 2 | 1 (1) | 4 | 6 (1) | 1 | – | 3 | 19 (56) |
| Neurocortical | – | – | – | – | – | – | – | – | 0 (0) |

*Patients initially treated as a higher dose level; **grade 1, mild; grade 2, moderate; grade 3, severe.

was observed in three courses. Diarrhoea grades 1 and 2 occurred in 8% of courses, being severe (grade 4) in only one course at dose level VA. Mucositis grades 1 and 2 were documented in 24% of courses, being severe in one course at dose level VIA and in one course at dose level IIIB. The incidence of gastrointestinal toxicity appeared to be higher in schedule B. Asthenia occurred at all dose levels and was not related to the schedule. The docetaxel–ifosfamide combination induced a mild, sensory neuropathy grade 1–2 in 56% of patients. However, no neurocortical toxicities such as ifosfamide-induced encephalopathy were observed. Docetaxel-related toxicities such as skin and nail changes and oedema were mild and never a reason to stop therapy. They occurred in 38%, 24% and 15% of patients respectively. Hypersensitivity reactions were mild to moderate and consisted of flushing and dyspnoea in some patients. They only occurred in 5% of courses but were more frequent in schedule B.

*© Cancer Research Campaign 1998*

### RESPONSES

A histologically proven complete response (CR) was achieved in a patient with a soft tissue sarcoma treated at dose level II. Partial responses were observed in cancer of unknown primary and in non-small-cell lung cancer, one patient each.

### DISCUSSION

Docetaxel is a new antimicrotubule agent that has already demonstrated activity in a wide variety of solid tumours and was registered for use in advanced breast cancer in 1995. Ifosfamide is an alkylating agent that is active when administered orally in non-small-cell lung cancer, testicular cancer, breast cancer and sarcoma. Because of the partly overlapping toxicity profiles and their activity against a wide range of solid tumours it was considered of interest to pursue a combination regimen of these two drugs. This phase I study was performed to assess the feasibility of the combination, to determine the MTD and the side-effects and to evaluate if toxicity is drug sequence dependent.

No phase I studies on the combination docetaxel–ifosfamide had been performed previously. The major toxicity of the combination was granulocytopenia grades 3 and 4, which occurred in 89% of all courses and appeared to be ifosfamide dose dependent. Neutropenia grade 4 associated with fever occurred in 17% of all courses and was more common at the highest dose levels. The DLT for schedule A was neutropenic fever at a dose of 85 mg $m^{-2}$ of docetaxel and 5 g $m^{-2}$ of ifosfamide (dose level VIA). The DLT for schedule B was neutropenic fever at a dose level of 75 mg $m^{-2}$ of docetaxel and 4 g $m^{-2}$ of ifosfamide (dose level IVB), which was the initial dose tested. This observation cannot be explained by a difference in haematological toxicity between schedule A and B or by a difference in patient selection. As this made clear that there was no obvious advantage of this schedule compared with schedule A, no further patients have been studied.

The most common non-haematological toxicities were nausea, vomiting, mucositis and diarrhoea, most of them being mild. Schedule B appeared to induce more gastrointestinal toxicity than schedule A, for which no explanation can be given. In 19 out of 34 evaluable patients (56%) the docetaxel–ifosfamide combination induced a sensory neuropathy grade 1–2, whereas no neurocortical toxicity was observed. The incidence of sensory neuropathy of the combination was slightly higher than the incidence reported for docetaxel as a single agent (49%) (Hilkens et al, 1996). In a phase I study combining paclitaxel and cisplatin (Rowinsky et al, 1991) and in a phase I study on the docetaxel–cisplatin combination (Hilkens et al, 1997; Pronk et al, 1997), the incidence of sensory neuropathy of the combination was higher than that reported for docetaxel as a single agent (Hilkens et al, 1996). It was suggested that these agents act synergistically in producing neurotoxicity.

The incidence of docetaxel related toxicities such as hypersensitivity reactions, nail and skin toxicity and oedema was relatively low and never a reason for treatment discontinuation. In this study, all patients received premedication consisting of dexamethasone or methylprednisolone, which might account for the low incidence of the above-mentioned side-effects.

Anti-tumour responses were seen in a variety of tumour types at all dose levels. Of note, was the histologically proven complete response in a patient with soft tissue sarcoma.

Based upon the data obtained in this phase I study the recommended dose for phase II studies will be 75 mg $m^{-2}$ of docetaxel combined with 5 g $m^{-2}$ of ifosfamide. Schedule A is advocated for further treatment because this schedule is more manageable and seems to induce less gastrointestinal toxicity than schedule B.

### REFERENCES

Aamdal S, Wolff I, Kaplan S, Paridaens R, Kerger R, Schachter J, Wanders J, Franklin H and Verweij J (1994) Taxotere in advanced malignant melanoma: A phase II trials of the EORTC Clinical Trials Group. *Eur J Cancer* **30A**: 1061–1064

Aapro MS, Zulian G, Alberto P, Bruno R, Oulid-Aissa D and Le Bail N (1992) Phase I and pharmacokinetic study of RP 56976 in a new ethanol-free formulation of taxotere. *Ann Oncol* **3** (suppl. 5): 208

Bissery MC, Guenard D, Gueritte-Voegelein F and Lavelle F (1991) Experimental antitumor activity of taxotere (RP 56976, NSC 628503), a taxol analogue. *Cancer Res* **51**: 4845–4852

Bisset D, Setanoians A, Cassidy J, Graham MA, Chadwick GA, Wilson P, Auzannet V, Le Bail N, Kaye SB and Kerr DJ (1993) Phase I and pharmacokinetic study of taxotere (RP 56976) administered as a 24-hour infusion. *Cancer Res* **53**: 523–527

Ten Bokkel-Huinink WW, Prove AM, Piccart M, Steward W, Tursz T, Wanders J, Franklin H, Clavel M, Verweij J, Alakl M, Bayssas M and Kaye SB (1994) A phase II trial with docetaxel (taxotere) in second line treatment with chemotherapy for advanced breast cancer. A study of the EORTC Early Clinical Trials Group. *Ann Oncol* **5**: 527–532

Brundage MD, Pater JL and Zee B (1993) Assessing the reliability of two toxicity scales: implications for interpretating toxicity data. *J Natl Cancer Inst* **85**: 1138–1148

Burris H, Irvin R, Kuhn J, Kalter S, Smith L, Shaffer D, Fields S, Weiss G, Eckardt J, Rodriguez G, Rinaldi D, Wall J, Cook G, Smith S, Vreeland F, Bayssas M, Le Bail N and Von Hoff D (1993) Phase I clinical trial of taxotere administered as either a 2-hour or 6-hour intravenous infusion every 3 weeks. *J Clin Oncol* **11**: 950–958

Catimel G, Verweij J, Mattijsen V, Hanauske A, Piccart M, Wanders J, Franklin H, Le Bail N, Clavel M and Kaye SB (1994) Docetaxel (taxotere): an active drug for the treatment of patients with advanced squamous cell carcinoma of the head and neck. *Ann Oncol* **5**: 533–537

Cerny T, Kaplan S, Pavlidis N, Schöffski P, Epelbaum R, Van Meerbeek J, Wanders J, Franklin HR and Kaye S, for the ECTG (1994) Docetaxel (taxotere) is active in non-small cell lung cancer: A phase II trial of the EORTC-Clinical Trials Group. *Br J Cancer* **70**: 384–387

Chevallier B, Fumoleau P, Kerbrat P, Dieras V, Roche H, Krakowski I, Azli N, Bayssas M, Lentz MA and Van Glabbeke M (1995) Docetaxel is a major cytotoxic drug for the treatment of advanced breast cancer: a phase II trial of the Clinical Screening Cooperative Group of the European Organization for Research and Treatment of Cancer. *J Clin Oncol* **13**: 314–322

Denis JN, Greene AE, Guenard D, Gueritte-Voegelein F, Mangatal L and Potier PA (1988) A highly efficient practical approach to natural taxol. *J Am Chem Soc* **110**: 5917–5919

Ettinger DS. (1989). Ifosfamide in the treatment of non-small cell lung cancer. *Semin Oncol* **16** (suppl. 5): 31–38

Extra JM, Rousseau F, Bruno R, Clavel M, Le Bail N, Marty M (1993) Phase I and pharmacokinetic study of taxotere (RP56976; NSC 628503) given as a short intravenous infusion. *Cancer Res* **53**: 1037–1042

De Forni M, Rougier P, Adenis A, Ducreux M, Djazouli K, Adams D, Clouet P, Blanc C, Bayssas M, Bonneterre J and Armand JP (1994) Phase II study of taxotere in locally advanced and/or metastatic pancreatic cancer. *Ann Oncol* **5** (suppl. 5): 509

Fossella FV, Lee JS, Shin DM, Calayag M, Huber M, Perez-Soler R, Murphy WK, Lippman S, Benner S, Glisson B, Chasen M, Ki Hong W and Raber M (1995) Phase II study of docetaxel for advanced or metastatic platinum-refractory non-small cell lung cancer. *J Clin Oncol* **13**: 645–651

Gueritte-Voegelein F, Guenard D, Lavelle F, Le Goff MT, Mangatal L and Potier P (1991) Relationships between the structure of taxol analogues and their antimitotic activity. *J Med Chem* **34**: 992–998

Harrison S, Dykes D, Sheperd RV, Griswold DP and Bissery MC (1992) Response of human tumor xenografts to taxotere. *Proc Am Assoc Cancer Res* **33**: 526

Hilkens PHE, Verweij J, Stoter G, Vecht CHJ, Van Putten WLJ and VD Bent MJ (1996) Peripheral neurotoxicity induced by docetaxel. *Neurology* **46**: 104–108

Hilkens PHE, Pronk LC, Verweij J, Vecht CHJ, Van Putten WLJ and VD Bent MJ (1997) Peripheral neuropathy induced by combination chemotherapy of docetaxel and cisplatin. *Br J Cancer* **75**: 417–422

© Cancer Research Campaign 1998

Van Hoesel QGCM, Verweij J, Catimel G, Clavel M, Kerbrat P, Van Oosterom AT, Kerger J, Tursz T, Van Glabbeke M, Van Pottelsberghe C, Le Bail N and Mouridsen H, for the EORTC Soft Tissue and Bone Sarcoma Group (1994) Phase II study with docetaxel (taxotere) in advanced soft tissue sarcomas of the adult. *Ann Oncol* **5**: 539–542

Hoffmann W, Weidmann B, Migeod F, Könner J and Seeber S (1990) Epirubicin and Ifosfamide in patients with refractory breast cancer and other metastatic solid tumours. *Cancer Chemother Pharmacol* **26** (suppl.): 69–70

Kaye SB, Mead GM, Fossa S, Cullen M, De Wit R, Bodrogi I, Van Groeningen C, Sylvester R, Stenning S, Vermeylen K, Lallemand E and De Mulder P, From the MRC and EORTC Data Centres, Cambridge and Brussels (1995) An MRC/EORTC randomised trial in poor prognosis metastatic teratoma, comparing BEP with BOP-VIP. *Proc ASCO*, **14**: 246

Miller VA, Rigas JR, Francis PA, Grant SC, Pisters KMW, Venka Traman ES, Woolley K, Heelan RT and Kris MG (1995) Phase II trial of a 75 mg/m$^2$ dose of docetaxel with prednisone premedication for patients with advanced non-small cell lung cancer. *Cancer* **75**: 968–972

Nicoletti MI, Massazza G, Abbott BJ, D'Incalci M and Giavazzi R (1992) Taxol and taxotere antitumor activity on human ovarian carcinoma xenografts. *Proc Am Assoc Cancer Res* **33**: 519

Pazdur R, Newman RA, Newman BM, Fuentes A, Benvenuto J, Bready B, Moore D, Jaiyesimi I, Vreeland F, Bayssas MMG and Raber MN (1992) Phase I trial of taxotere: five day schedule. *J Natl Cancer Inst* **84**: 1781–1788

Piccart MJ, Klijn J, Mauriac L, Nooij M, Paridaens R, Coleman R, Selleslags J, Van Vreckem A and Van Glabbeke M (1994) Weekly docetaxel with or without prophylactic steroids as 2nd line treatment for metastatic breast cancer: a randomized trial of the EORTC Breast Cancer Study Group. *Ann Oncol* **5** (suppl 8): 27

Pinedo HM and Verweij J (1986) The treatment of soft tissue sarcomas with focus on chemotherapy. A review. *Radiother Oncol* **5**: 193–205

Pronk LC, Stoter G and Verweij J (1995) Docetaxel (Taxotere): single agent activity, development of combination treatment and reducing side effects. *Cancer Treatment Rev* **21**: 463–478

Pronk LC, Schellens JHM, Planting ASTH, VD Bent MJ, VD Burg Mel, De Boer-Dennert M, Ma J, Blanc C, Harteveld M, Bruno R, Stoter G and Verweij J (1997) A phase I and pharmacological study of docetaxel and cisplatin in patients with advanced solid tumors. *J Clin Oncol* **15**: 1071–1079

Ringel I and Horwitz SB (1991) Studies with RP 56976 (taxotere): a semi-synthetic analog of taxol. *J Natl Cancer Inst* **83**: 288–291

Rowinsky EK and Donehower RC (1991) The clinical pharmacology and use of antimicrotubule agents in cancer chemotherapeutics. *Pharmac Ther* **52**: 35–84

Rowinsky EK, Gilbert MR, McGuire WP, Noe DA, Grochow LB, Forastiere AA, Ettinger DS, Lubejko BG, Clark B, Sartorius SE, Cornblath DR, Hendricks CB and Donehower RC (1991) Sequences of taxol and cisplatin: A phase I and pharmacologic study. *J Clin Oncol* **9**: 1692–1703

Schrijvers D, Wanders J, Dirix L, Prove A, Vonck I, Van Oosterom A and Kaye S (1993) Coping with toxicities of docetaxel (Taxotere). *Ann Oncol* **4**: 610–611

Seidman AD, Hudis C, Crown JPA, Balmaceda C, Lebwohl D, Currie V, Gilewski T, Hakes T, Robles M, Klem K, Lepore J and Norton L (1993) Phase II evaluation of taxotere as initial chemotherapy for metastatic breast cancer. *Proc ASCO* **12**: 63

Sulkes A, Smyth J, Sessa C, Van Oosterom AT, Vermorken J, Kaye SB, Le Bail N and Verweij J for the EORTC Early Clinical Studies Group (1994) Docetaxel (taxotere) in advanced gastric cancer: results of a phase II clinical trial. *Br J Cancer* **70**: 380–383

Tomiak E, Piccart MJ, Kerger J, Lips S, Awada A, De Valeriola D, Ravoet C, Lossignol D, Sculier JP, Auzannet V, Le Bail N, Bayssas M and Klastersky J (1993) Phase I study of taxotere (RP 56976, NSC 628503) administered as a one hour intravenous infusion on a weekly basis. *J Clin Oncol* **12**: 1458–1467

Trudeau M, Eisenhauer E, Lofters W, Norris B, Muldal A, Letendre F, Vandenburg T and Verma S (1993) Phase II study of taxotere as first line chemotherapy for metastatic breast cancer: A National Cancer Institute of Canada Clinical Trial Group study. *Proc ASCO* **12**: 64

Valero V, Esparza L, Holmes F, Walters R, Fraschini G, Theriault R, Dhingra K, Buzdar A, Bellet R, Slattery A, Pazdur R, Raber M, Bayssas M and Hortobagyi G (1993) Phase II study of taxotere in refractory metastatic breast cancer. *Proc ASCO* **12**: 96

WHO handbook for reporting results of cancer treatment. Geneva, *WHO Offset Publication 1979* **48**: pp. 22–26

© Cancer Research Campaign 1998