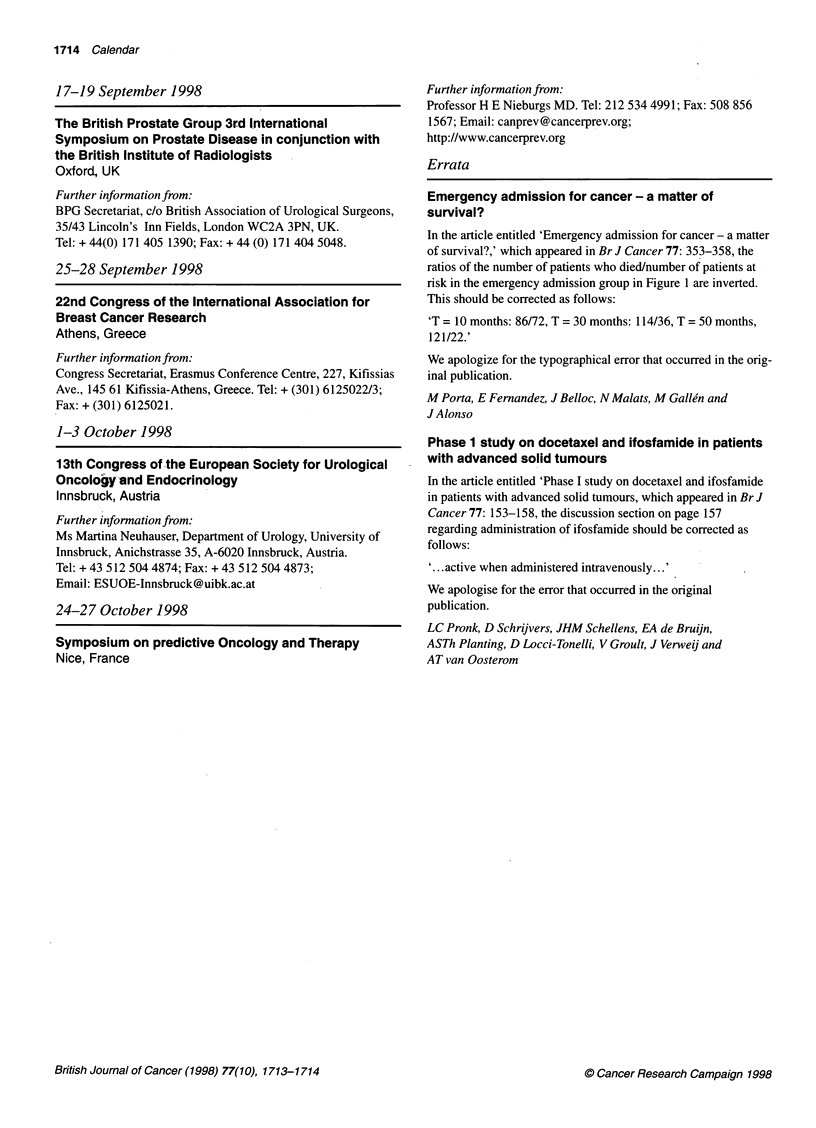# Phase 1 study on docetaxel and ifosfamide in patients with advanced solid tumours

**Published:** 1998-05

**Authors:** 


### Phase 1 study on docetaxel and ifosfamide in patients with advanced solid tumours

In the article entitled 'Phase I study on docetaxel and ifosfamide in patients with advanced solid tumours, which appeared in *Br J Cancer* **77**: 153–158, the discussion section on page 157 regarding administration of ifosfamide should be corrected as follows:

'...active when administered intravenously...'

We apologise for the error that occurred in the original publication.

*LC Pronk, D Schrijvers, JHM Schellens, EA de Bruijn, ASTh Planting, D Locci-Tonelli, V Groult, J Verweij and AT van Oosterom*

*© Cancer Research Campaign 1998*